# Pioglitazone-Loaded Nanostructured Hybrid Material for Skin Ulcer Treatment

**DOI:** 10.3390/ma13092050

**Published:** 2020-04-28

**Authors:** Agnieszka Rojewska, Anna Karewicz, Karolina Karnas, Karol Wolski, Mateusz Zając, Kamil Kamiński, Krzysztof Szczubiałka, Szczepan Zapotoczny, Maria Nowakowska

**Affiliations:** Faculty of Chemistry, Jagiellonian University, Gronostajowa 2, 30-387 Kraków, Poland

**Keywords:** alginate, hydroxypropyl cellulose, pioglitazone hydrochloride, bacterial nanocellulose, wound healing, drug release

## Abstract

Pioglitazone, a popular antidiabetic drug, which was recently shown to be effective in the treatment of skin ulcers, was successfully encapsulated in polysaccharide nanoparticles and used as a bioactive component of the wound-dressing material based on modified bacterial nanocellulose. Alginate and hydroxypropyl cellulose were used as a matrix for the nanoparticulate drug-delivery system. The matrix composition and particles’ size, as well as drug encapsulation efficiency and loading, were optimized. Pioglitazone hydrochloride (PIO) loaded particles were coated with chitosan introduced into the crosslinking medium, and covalently attached to the surface of bacterial nanocellulose functionalized with carboxyl groups. PIO was released from the surface of the hybrid material in a controlled manner for 5 days. Preliminary cytotoxicity studies confirmed safety of the system at PIO concentrations as high as 20 mg/mL. The obtained hybrid system may have potential application in the treatment of skin ulcers e.g., in diabetic foot.

## 1. Introduction

Diabetic foot ulcer is a main complication of diabetes mellitus. It significantly decreases the quality of life of diabetic patients and may lead to very serious complications, including leg amputation. In addition to the systemic treatment with antibiotics, wound-healing dressings are a crucial element of the therapy. Among wound-healing materials used in diabetic foot syndrome hydrogels and hydrocolloids constitute an important and widely used class of materials. Hydrogels and hydrogel sponges allow to prevent infection, maintain moist environment, and absorb exudates [1]. They may also serve as bioactive material designed to release active agents which stimulate healing process. Hydrogels used for wound protection are usually made of natural polymers, often with special beneficial properties, such as responsiveness to the environmental changes, or ability to deliver drugs in a controlled manner.

Pioglitazone has been used for years to treat diabetes mellitus type 2 by reducing insulin resistance and decreasing gluconeogenesis in the liver. It belongs to the group of the peroxisome proliferator-activated receptor gamma (PPAR-γ) agonists. PPARs regulate gene expression, cell proliferation, differentiation, and apoptosis; they also play an important role in the immunomodulation. PPARs modulate also various skin functions and their important role in wound repair was first indicated by Wahli in 2002 [2]. Pioglitazone, PPAR-γ specific ligand, was shown to modulate the inflammatory processes and expression of various cytokines. The protective action of pioglitazone in the wound-healing was first noticed against gastric mucosal damage [3]. Lahiri et al. [4] reported that pioglitazone suppressed the expression of pro-inflammatory factors, such as tumor necrosis factor α (TNF-α) or interleukin 1β (IL-1β), in the healing of chronic gastric ulcer, and suggested the mediatory role of the interaction and combined upregulation of PPAR-γ and glucocorticoid receptor in this process. Similarly, Moezi et al. [5] have confirmed the gastroprotective influence of this drug on the stomach ulcers of cholestatic rats, indicating the TNF-α downregulation, constitutive nitric oxide synthase (cNOS) induction, and inducible nitric oxide synthase (iNOS) inhibition as most probable reasons. Konturek et al. [6] showed that pioglitazone was also effective in gastric ulcer treatment of diabetic rats, although its action was attenuated in comparison to non-diabetic animals. In 2015 Gupta et al. [7] pointed out PPAR agonists as a ‘future’ therapeutic in dermatology and, given their importance in all stages of wound healing, underlined their role as a possible remedy to the non-healing wounds. The positive effect of pioglitazone on the skin ulcer in diabetic mice was recently confirmed by Sakai et al. [8]. The authors showed that hydrogel-encapsulated pioglitazone stimulated wound-healing by downregulating iNOS, macrophage inflammatory protein 1 (MIP-1), transforming growth factor beta (TGF-β) and promoting M2 macrophages differentiation. The latter is of a special importance, as it was shown that the switch of macrophages from pro-inflammatory type to the healing-associated phenotype (M2) is impaired in diabetes, being one of the main reasons of chronic wounds [9]. Recently the role of pioglitazone in decreasing the level of metalloproteinase-9 (MMP-9) was also indicated [10,11]. MMP-9 is known to impair diabetes-associated wound healing processes.

Other PPAR-γ agonists were also proposed in wound repair applications. Silva et al. [12] have proposed a new thiazolidine compound, GQ-11, entrapped in Pluronic F-127^®^ gel as effective wound-healing agent. The comparative studies performed for GQ-11 and pioglitazone showed that both compounds were effective in downregulating pro-inflammatory factors: IL-6, TNF-α, and monocyte chemoattractant protein 1 (MCP-1), upregulating IL-10, and improving re-epithelization. GQ-11, being partial PPAR-γ/α agonist, showed slightly better performance, with increased collagen deposition and re-epithelization. One must, however, remember that, unlike pioglitazone, GQ-11 is not available commercially, or FDA approved.

Pioglitazone was also entrapped in hydrogel scaffold composed of chitosan and hydroxypropyl methyl cellulose and tested as wound-healing material in vitro and in vivo in diabetic model [13]. It was shown that pioglitazone-containing hydrogel significantly increased wound contraction, decreased MMP-9 and TNF-α levels, and increased vascular endothelial growth factor (VEGF) level. It resulted in reduced inflammation and enhanced wound-healing. The proposed scaffold was obtained by mixing both components and liophylization, with no cross-linking process. No degradation studies were done, but pioglitazone release was observed only for 24 h, and it may be expected that material has low long-term stability.

Polysaccharides, such as cellulose, have been used with success in the treatment of non-healing chronic wounds [14]. Cellulose is widely used as a wound-dressing material, mostly because it is vastly abundant in nature and exhibits excellent properties for the application. Bacterial nanocellulose (BNC) is most widely used due to its high purity, high water binding capacity, high permeability, and good shape retention [15,16]. It is commercially available, relatively cheap, and is characterized by a long-term stability. BNC network may be easily modified either chemically [17] or physically [18].

For all these reasons we have decided to use BNC as a base for a bioactive material designed to improve wound-healing in diabetic patients. Hydrochloride salt of pioglitazone (PIO) was introduced to the surface of BNC. Pioglitazone is sparingly soluble in water (intrinsic solubility of pioglitazone in water at 20 °C is as low as 0.1 µg/mL [19]) and, although the solubility of PIO is higher, the salt still needs to be delivered using a carrier system. PIO was therefore first encapsulated in the hydrogel nanoparticles, which additionally provided a prolonged, controlled release of the drug, and then introduced onto the surface of BNC. Alginate (ALG) was chosen to entrap PIO, mainly due to its ability to form hydrogels in an easy and biologically safe crosslinking process, without the need of using solvents or harsh conditions. Hydroxypropyl cellulose (HPC) was added in order to strengthen the relatively weak alginate gel. PIO was incorporated into the hydrogel particles during their formation. To covalently bind PIO-containing nanoparticles to BNC, we have first modified cellulose surface with carboxyl groups (mBNC). PIO-containing nanoparticles were then coated with chitosan, and covalently attached to mBNC through the formation of amide bonds. The chitosan coating provided additional, outer layer of nanoparticles, which hindered PIO release, allowing for a slow, gradual delivery of the drug. The commercially available BNC sheets decorated with biocompatible, containing PIO polysaccharide nanoparticles allowing the slow drug release constitute an attractive novel material for the effective treatment of chronic wounds and skin ulcers in diabetic patients.

## 2. Materials and Methods

### 2.1. Materials

Bionanocellulose modified with carboxyl groups (mBNC) was kindly provided by Biovico^®^ company—the modified BNC had 6.07 ± 0.42 nmol of carboxylic groups per 1 mm^2^ of mBNC surface [20]. Hydroxypropyl cellulose (HPC, M_v_ ≈ 80,000 g/mol, Sigma-Aldrich, Poznań, Poland), alginic acid sodium salt (ALG, medium molecular weight, from brown algae, Sigma-Aldrich, Poznań, Poland; Mv = 260,000 g/mol, M/G ratio = 1.20), chitosan (low molecular weight, Sigma-Aldrich, Poznań, Poland; Mv = 120,000 g/mol, degree of deacetylation DDA = 79%), pioglitazone hydrochloride (PIO) (≥98% (HPLC) Sigma-Aldrich, Poland), Pluronic^®^ P103 (PEO/PPO block copolymer, M_W_ = 4950 g/mol, BASF), Tween^®^ 85 (Polyoxyethylenesorbitan Trioleate, M_W_ = 1839 g/mol, Sigma-Aldrich, Poland, calcium chloride (p.a. Fluka, Poland), acetic acid (≥99%, Sigma-Aldrich, Poland), 1-ethyl-3-(3-dimethylaminopropyl)carbodiimide (EDC, Sigma-Aldrich, commercial grade, powder), N-hydroxysuccinimide (NHS, 98%, Sigma-Aldrich, Poznań, Poland), methanol (gradient grade for liquid chromatography, Sigma-Aldrich, Poznań, Poland), ethanol (gradient grade for liquid chromatography, Sigma-Aldrich, Poznań, Poland) were used as received. Mouse Embryonic Fibroblasts 3T3-L1 line was obtained from ATTC, Poland. Phosphate buffered saline tablets (PBS), Dulbecco’s Modified Eagle’s Medium—high glucose (DMEM) and Cell Proliferation Kit II (XTT) were purchased from Sigma-Aldrich, Poznań, Poland; HyClone trypsin-EDTA, HyClone Penicillin-Streptomycin Solution and HyClone Research Grade Fetal Bovine Serum, South American Origin (FBS) were purchased from Symbios.

### 2.2. PIO Solubilization-Turbidimetric Measurements

Although better soluble than pioglitazone, hydrochloride salt still needed to be introduced into the hydrogel matrix by solubilizing it in the surfactant solution. Turbidimetric measurements were performed in order to determine the maximal amount of PIO that could be solubilized in 1 mL of the aqueous solution of surfactant (Pluronic^®^ P103 or Tween^®^ 85). PIO dissolved in ethanol (0.5 mg/mL) was added in small portions (20 µL) to the 1 mL of the aqueous solution of either Pluronic^®^ P103 (6.1 × 10^−4^ M) or Tween^®^ 85 (6 × 10^−3^ M) in the quartz cuvette. After each addition of PIO solution to the surfactant solution the system was thoroughly mixed using magnetic stirrer, allowed to equilibrate and then its optical density was measured at 450 nm (where no absorption was observed from any of the components) using the HP 8452 Hewlett Packard spectrophotometer. The experiment was continued until the scattering was observed.

### 2.3. Synthesis of Hydrogel Nanoparticles Containing Surfactant Micelles with Solubilized PIO (NP-PIO)

To facilitate the encapsulation of the drug into the hydrophilic hydrogel nanospheres, 2 mL of the 0.5 mg/mL ethanolic solution of PIO was added dropwise to 8 mL of 6.1 × 10^−4^ M solution of Pluronic^®^ P103 under continuous stirring. A total of 12.5 mg of HPC and 50 mg of ALG were then added to the solution. After stirring for 1 h at room temperature, the resulting mixture was introduced to the syringe and injected via a needle using a syringe pump (World Precision Instruments, Aladdin-1000, 0.05 mL/min) to a 50 mL solution of cross-linking agent (0.2 M calcium chloride dissolved in 1% *w*/*v* chitosan solution in 1% *w*/*v* acetic acid) under continuous stirring. The obtained nanoparticles (NP-PIO) were filtered off on a fritted glass funnel (11 G-4), washed with distilled water, and dried at room temperature.

### 2.4. Release Profiles Entrapment Efficiency and Loading Capacity Determination

Four 10 mg samples of the NP-PIO particles were weighted, and each placed in a 10 mL centrifuge tube. Then, 1 mL of a mixture of 10 mM phosphate-buffered saline (PBS) aqueous solution (pH = 7.4) and methanol (*v*/*v* = 1:1) was added to each tube. The samples were then incubated at 37 °C (IKA, a KS 3000 incubator) with steady agitation (140 rpm). After predefined time intervals all samples were centrifuged at 10,000 rpm for 10 min and then the supernatants were collected. The new portion of the PBS-methanol mixture was added to the particles in each tube, and the samples were placed back in the incubator. The concentration of the drug in each of the collected supernatants was quantified spectrophotometrically by measuring their absorbance at 267 nm (the maximum of PIO absorption). In order to determine the total amount of PIO loaded into the particles, the amount of PIO remaining in the particles after 5 days of release studies was also measured. After the release studies were complete, 1 mL of PBS-methanol mixture was added to each NP-PIO sample and they were sonicated for 90 min, which resulted in a complete disintegration of the particles. The samples were centrifuged, and PIO concentration was measured in each supernatant in the same way it was done during release studies. The calculations of PIO concentration were done based on the calibration curve obtained for the standard solutions of PIO in PBS-methanol mixture (*v*/*v* = 1:1). High linearity was achieved in the required concentration range with the correlation coefficient value R^2^ = 0.999. Entrapment efficiency (EE [%]) and loading efficiency (LE [%]) were calculated from the following equations:

(1) $$\mathrm{EE}\;\left[\%\right]=\frac{\mathrm{total}\;\mathrm{weight}\;\mathrm{of}\;\mathrm{PIO}\;\mathrm{in}\;\mathrm{the}\;\mathrm{obtained}\;\mathrm{particles}\;}{\mathrm{weight}\;\mathrm{of}\;\mathrm{PIO}\;\mathrm{used}\;\mathrm{in}\;\mathrm{the}\;\mathrm{synthesis}}\times 100$$

(2) $$\mathrm{LE}\;\left[\%\right]=\frac{\mathrm{weight}\;\mathrm{of}\;\mathrm{PIO}\;\mathrm{encapsulated}\;\mathrm{in}\;\mathrm{the}\;\mathrm{sample}\;}{\mathrm{weight}\;\mathrm{of}\;\mathrm{the}\;\mathrm{sample}}\times 100$$

### 2.5. Deposition of NP-PIO/Ch Particles on Modified Bacterial Nanocellulose (mBNC)

To deposit chitosan-coated nanospheres on the surface of bacterial nanocellulose modified with carboxyl groups (mBNC), a procedure described and optimized in our previous work was applied [21]. A 4 cm^2^ patch of mBNC was immersed in an acetate buffer (pH = 5.5) at room temperature. N-hydroxysuccinimide (NHS) (0.025 M) and 1-ethyl-3-(3-dimethylaminopropyl)carbodiimide EDC (0.008 M) were then added and the sample was continuously shaken at 150 rpm for 2 h. Activated mBNC was then rinsed with deionized water and incubated with the suspension of the nanospheres (100 mg) in phosphate buffer (pH = 7.4) for 30 min. The unbound particles were then removed by a thorough washing with deionized water. The obtained samples of mBNC with attached particles were then dried in the air.

**Figure 1:**
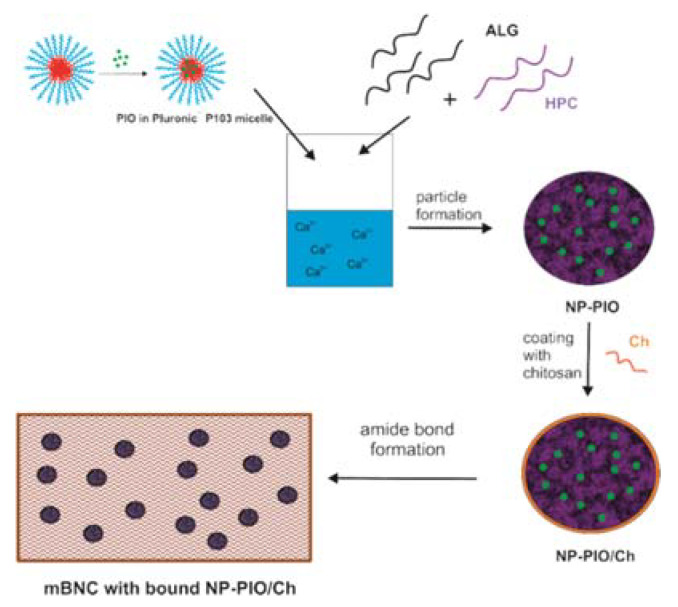
Synthesis of hybrid nanostructured material for skin ulcer treatment.

The schematic presentation of the whole process of the synthesis of nanostructured hybrid material was shown in Figure 1.

### 2.6. Scanning Electron Microscopy (SEM) Analysis

SEM analysis was carried out using a PhenomWorld Pro scanning electron microscope (Waltham, MA, USA). NP-PIO/Chit particles were dried at room temperature on a watch glass, and then the obtained material was placed on a carbon tape. mBNC with the particles was stretched flat on a glass slide, dried in vacuum, and placed on a carbon tape.

### 2.7. AFM Measurements

Atomic force microscope (AFM) images were obtained using a Dimension Icon AFM microscope (Bruker, Santa Barbara, CA, USA) working in the PeakForce Tapping (PFT) and QNM^®^ modes with standard silicon cantilevers for measurements in the air (nominal spring constant of 0.4 N/m).

### 2.8. Fourier-Transform Infrared Spectroscopy (FTIR)

FTIR spectra were recorded using a Nicolet iS10 FT-IR spectrometer with an Attenuated Total Reflectance (ATR) attachment (SMART iTX) (Waltham, MA, USA).

### 2.9. X-ray Diffraction (XRD) Measurements

X’Pert PRO MPD diffractometer (Malvern, United Kingdom) by PANalytical with a Bragg–Brentano geometry was used for all XRD measurements. A copper x-ray sealed tube was used as the radiation source. A graphite monochromator was applied to select only Cu Kα (1.540598 Å—Kα1 and Kα2-1.544426 Å) radiation. 

### 2.10. Differential Scanning Calorimetry (DSC) Measurements

DSC measurements were carried out on a Mettler Toledo DSC 821e model (Columbus, OH, USA). The accurately weighed sample was placed in an aluminum pan, and an empty aluminum pan was used as a reference. The experiment was carried out in argon atmosphere at a scanning rate of 10 °C/min., in the range of 25 °C to 600 °C.

### 2.11. Fibroblasts Proliferation Studies

#### 2.11.1. Cell Culture

Ethical Statement: Mouse Embryonic Fibroblasts 3T3-L1 cell line was obtained from ATCC, Poland.

Mouse Embryonic Fibroblasts 3T3-L1 (MEFs) were incubated (37 °C, 90% humidity with 5% CO_2_) in a cell culture dishes containing DMEM supplemented with 100 U/mL of penicillin, 100 µg/mL of streptomycin, and 10% (*v*/*v*) of fetal bovine serum (FBS). The cells (at approximately 70% confluency) were washed twice with PBS solution (0.01 M phosphate buffer, 0.0027 M potassium chloride and 0.137 M sodium chloride, pH 7.4) and next sub-cultured with 1 mL of trypsin (0.25%) with EDTA (0.1%). After incubation for 3 min, 3 mL of DMEM (with 5% (*v*/*v*) FBS) were added and cell suspension was centrifuged for 5 min at 1250 *g*. The pellet was re-suspended in DMEM (5% (*v*/*v*) FBS).

#### 2.11.2. Proliferation

MEFs were seeded into a 48-well cell culture plates at 1.2 × 10^4^ (one-day test), 5.6 × 10^3^ (two-day test), 1.2 × 10^3^ (three-day test), and 3.0 × 10^3^ (four-day test) cells/well (DMEM, 5% FBS). After 15 h (one-day and two-day test) or 20 h (three-day and four-day test) of incubation (37 °C, 90% humidity, 5% CO_2_) the medium was replaced with fresh DMEM (5% FBS) containing particles at various concentrations. After a set time (1, 2, 3, or 4 days) the XTT assay was performed as follows: the medium was removed, the fresh DMEM (100 µL) and XTT mixture (50 µL) was added. After 2 h of incubation the absorbance at 460 nm was measured using Epoch™ 2 Microplate Spectrophotometer (Biotek Instruments, Inc, Winooski, VT, USA). The results for each concentration have been represented as a ratio of the mean absorbance for PIO-loaded particles (NP-PIO_B/Ch(15)) to the mean absorbance for “empty” particles (obtained according to the same methodology but without PIO added) with standard deviation (four replicates).

#### 2.11.3. Scratch Assay

MEFs were seeded into a 6-well cell culture plate at 6.2 × 10^4^ cells/well (DMEM, 10% FBS). After two days, a scratch in a cell monolayer was created in each well. Next the medium was removed, the fresh DMEM was added and the pictures have been taken at 4 different places along the scratch. After that, DMEM was replaced with the fresh DMEM with 5% FBS (control) or DMEM with empty or PIO-loaded nanoparticles (20 mg/mL, 5% FBS). After 11 h the medium was removed, fresh DMEM was added, and the pictures have been taken in the same places as before.

## 3. Results and Discussion

In the first stage of our studies we have tested and optimized the encapsulation of PIO in the hydrogel nanoparticles. The system was designed based on natural polysaccharides, ALG and HPC, which were highly biocompatible, as well as compatible with the proposed dressing, mBNC. The main role of the particulate system was to control the release rate of PIO. To ensure this control we have studied the influence of the composition of the hydrogel matrix and the type of the surfactant used to incorporate PIO into the hydrogel, as well as the impact of chitosan added to the crosslinking medium on the release profiles. The amount of the active agent encapsulated in the particles was also optimized.

ALG and HPC were used as the main components of the nanoparticles’ matrix. We have used the same components previously to encapsulate other biologically active agents [21,22,23] and we have found that the ratio of both polymers must be adjusted individually to each active compound being delivered. In the case of PIO, due to its moderate solubility in water, a surfactant (Pluronic^®^ P103 or Tween^®^ 85) micelles were used to facilitate the encapsulation of PIO in the relatively hydrophilic ALG/HPC matrix. Pluronic^®^ P103 (HLB = 9) is a bio-friendly polymer from the group of poloxamers, which is frequently used in pharmaceutical formulations. Tween^®^ 85 (HLB = 11) is a polysorbate, another nonionic surfactant used to stabilize aqueous formulations of medications, of a somewhat higher hydrophilic-lipophilic balance. To ensure the formation of micelles, the initial concentration of each surfactant was set to be ca. 100 times above its critical micelle concentration (CMC). The micelles containing PIO were then encapsulated in the polymeric hydrogel using extrusion method.

### 3.1. The Influence of the Matrix Composition on Encapsulation and Release of PIO

Based on the initial experiments, two ALG:HPC mixtures at the ratio 3:1 and 4:1 were selected to form the hydrogel matrix. Using these two compositions we have obtained the PIO-loaded particles and then we have performed their full characterization as drug-delivery systems, including their morphology and average size, PIO loading efficiency LE [%], entrapment efficiency EE [%], and release profile. Based on the obtained profiles we have determined the release time needed for each formulation to reach 50% release of PIO. The obtained results were summarized in Table 1.

**Figure 2:**
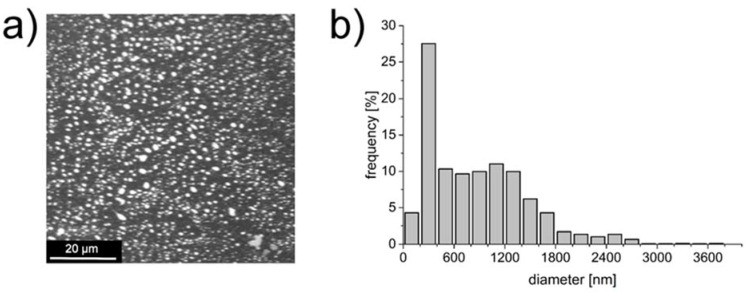
SEM image of the NP-PIO_B/Ch particles (**a**) and associated histogram (**b**).

The average diameter of each type of particles was obtained from the histograms calculated based on the SEM images. An example of SEM image and associated histogram are presented in Figure 2. In each case the spherical particles were obtained. The size distribution was rather broad, which is typical for extrusion method. The average sizes of both types of particles were slightly above 1 µm; the average diameter of the 3:1 particles (NP-PIO_A) was ca. 30% larger than for 4:1 particles (NP-PIO_B). The LE and EE values were higher for 4:1 particles than for 3:1 ones.

To increase the control over the release profile, both types of particles were additionally coated with a chitosan layer, to obtain NA-PIO_A/Ch and NP-PIO_B/Ch particles, respectively. The properties of the obtained systems were also presented in Table 1.

In the next step, we have examined the impact of the addition of chitosan during the formation of the particles to the crosslinking medium on the properties of resulting particles. The presence of chitosan in the cross-linking solution led to the reduction in particles’ size dispersity, which was more pronounced for the NP-PIO_B/Ch particles than for NP-PIO_A/Ch ones, and to the improvement of other important parameters, such as LE or EE. For NP-PIO_A/Ch particles LE was twice as high as for NP-PIO_A: the measured values were 1.04% and 0.50%, respectively, while the EE value increased from 43.20% to 72.85%. For the NP-PIO_B and NP-PIO_B/Ch particles the same tendency was clearly visible. Chitosan addition was previously reported to limit the rate of drug release from the alginate particles [24,25]. The carboxyl groups of alginate and the amine residues of chitosan interact to form the polyelectrolyte complex. The porosity of alginate beads is reduced upon complexation and, as a result, the leakage of the drug is reduced [26]. This leads to higher EE and LE values and a better controlled release rate.

**Figure 3:**
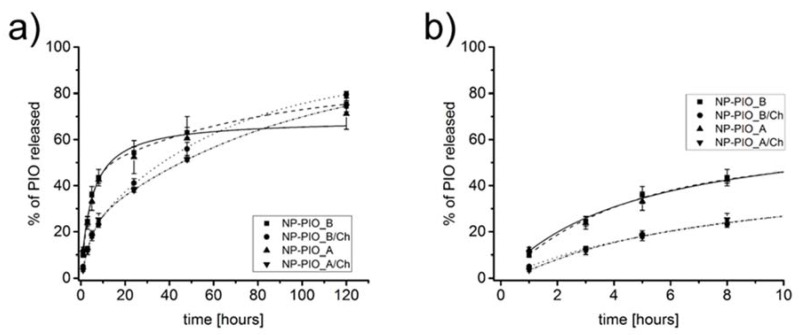
Full release profiles of pioglitazone hydrochloride (PIO) from the particulate delivery systems of different composition to a mixture of 10 mM phosphate-buffered saline (PBS) aqueous solution (pH = 7.4) and methanol (*v*/*v* = 1:1) at 37 °C (**a**) and first 10 h of the presented release profiles (**b**).

The release profiles of PIO from the obtained particulate systems of different composition were also studied. All profiles were obtained at 37 °C and pH = 7.4. Due to the low solubility of the drug and its slow release rate from the hydrogel system, we have decided to use a 1:1 (*v*/*v*) PBS (pH = 7.4): methanol mixture as a model release medium [27]. The ALG:HPC ratio had little effect on the release rate of PIO from the nanoparticles, although slight differences were noticed: in the case of the 4:1 system half of the entrapped drug was released in 24 h versus 25 h for the 3:1 system. Much more pronounced was the role of chitosan present in crosslinking medium. While for NP-PIO_A and NP-PIO_B 50% of the drug was released within 24 h, for the NP-PIO_A/Ch and NP-PIO_B/Ch half of the encapsulated PIO was released only after 45 h and 47 h, respectively. Thus, it can be concluded that chitosan present in the crosslinking solution is incorporated into the hydrogel matrix of the particles, significantly slowing down the release of the drug. The results of the release studies are presented in Figure 3. 

The comparison of LE, EE, and release profiles of all studied systems allowed us to select NP-PIO_B/Ch as the most promising system, as the particles were characterized by small size, relatively low size dispersity, the highest loading, high encapsulation efficiency, and favorable release profile with very limited burst effect and prolonged release up to 5 days.

### 3.2. Optimization of the PIO Loading

**Figure 4:**
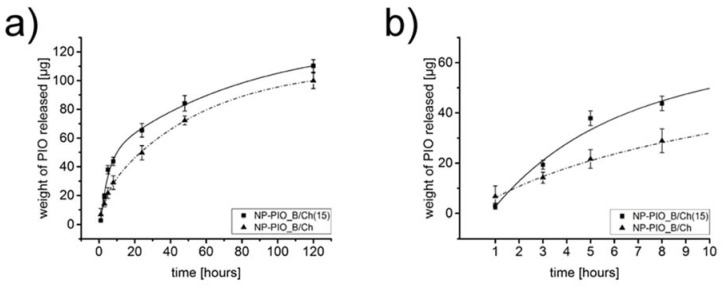
Full release profiles of PIO from NP-PIO_B/Ch, and NP-PIO_B/Ch(15) delivery systems to a mixture of 10 mM PBS aqueous solution (pH = 7.4) and methanol (*v*/*v* = 1:1) at 37 °C (**a**) and first 10 h of the presented release profiles (**b**).

In order to increase the loading efficiency of PIO in the proposed hydrogel system, we have made an attempt to increase the concentration of the drug in the micellar solution of surfactant. Two different, biocompatible surfactants of different structure and polarity, Tween^®^ 85 and Pluronic^®^ P103, were tested. Turbidimetric measurements were first used to establish the highest possible concentration of PIO in the micellar solution of each surfactant. The concentration of each surfactant was chosen so as it was 100 times higher than its CMC. The obtained PIO concentrations were found to be 0.15 mg/mL for Pluronic^®^ P103, and 0.19 mg/mL for Tween^®^ 85. Based on these results we have synthetized two additional systems using 0.15 mg/mL solution of PIO solubilized in either Pluronic^®^ P103 or Tween^®^ 85 micelles. It allowed us to compare two different surfactants while maintaining drug’s concentration as close as possible to the saturation limit, and without risking precipitation in either system. All other parameters of the procedure were kept unchanged. Particles prepared using Pluronic^®^ P103 (NP-PIO_B/Ch(15)) and Tween^®^ 85 (NP-PIO_B/Ch(15T)) were characterized and the obtained results were compared with the previously discussed systems in Table 1. Particles obtained using Tween^®^ 85 were of submicrometric size (ca. 700 nm), while the use of Pluronic^®^ P103 allowed to obtain nanoparticles with a diameter of ca. 150 nm. Considerable increase (20–23%) in the loading efficiency was observed for the optimized systems, as well as an increase in the encapsulation efficiency (by ca. 9–12%). An interesting change was observed in the release profiles, confirming the influence that the drug has on the structure of the hydrogel matrix (Figure 4)—the observed release for NP-PIO_B/Ch(15) was initially faster than for NP-PIO_B/Ch. After first 5 h, however, the release became comparable in both systems, and was nearly linear with time, confirming a very good control over the release process. PIO was released from NP-PIO_B/Ch(15) for up to 5 days.

**Figure 5:**
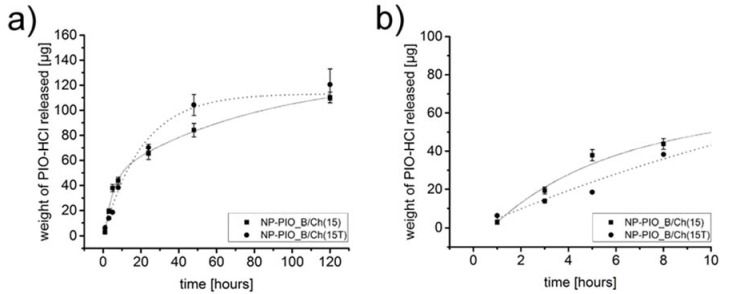
Full release profiles of PIO from NP-PIO_B/Ch(15), and NP-PIO_B(15T) delivery systems to a mixture of 10 mM PBS aqueous solution (pH = 7.4) and methanol (*v*/*v* = 1:1) at 37 °C (**a**) and first 10 h of the presented release profiles (**b**).

The comparison between the systems prepared using different surfactant revealed that in the case of system prepared using Pluronic^®^ P103 the drug was released slower during the first 10 h and the observed release profile was linear in this timeframe. For longer release times the opposite effect was observed—PIO was released slower from the particles obtained using Tween^®^ 85. The amount of PIO released from both systems after 5 days was, however, comparable (Figure 5). As more PIO is being released within the first 10 h, the particles obtained using Pluronic^®^ P103 seem a better choice. In addition, other parameters were slightly worse for the particles prepared in the presence of Tween^®^ 85 compared to the system where Pluronic^®^ P103 was used (larger size, lower LE, faster release—see Table 1). Thus, we have decided to discontinue further studies with the use of Tween^®^ 85 and the nanoparticulate system NP-PIO_B/Chit(15) was selected for further studies. 

### 3.3. Studies of the Interaction of PIO with Polymer Matrix of the Nanoparticles

**Figure 6:**
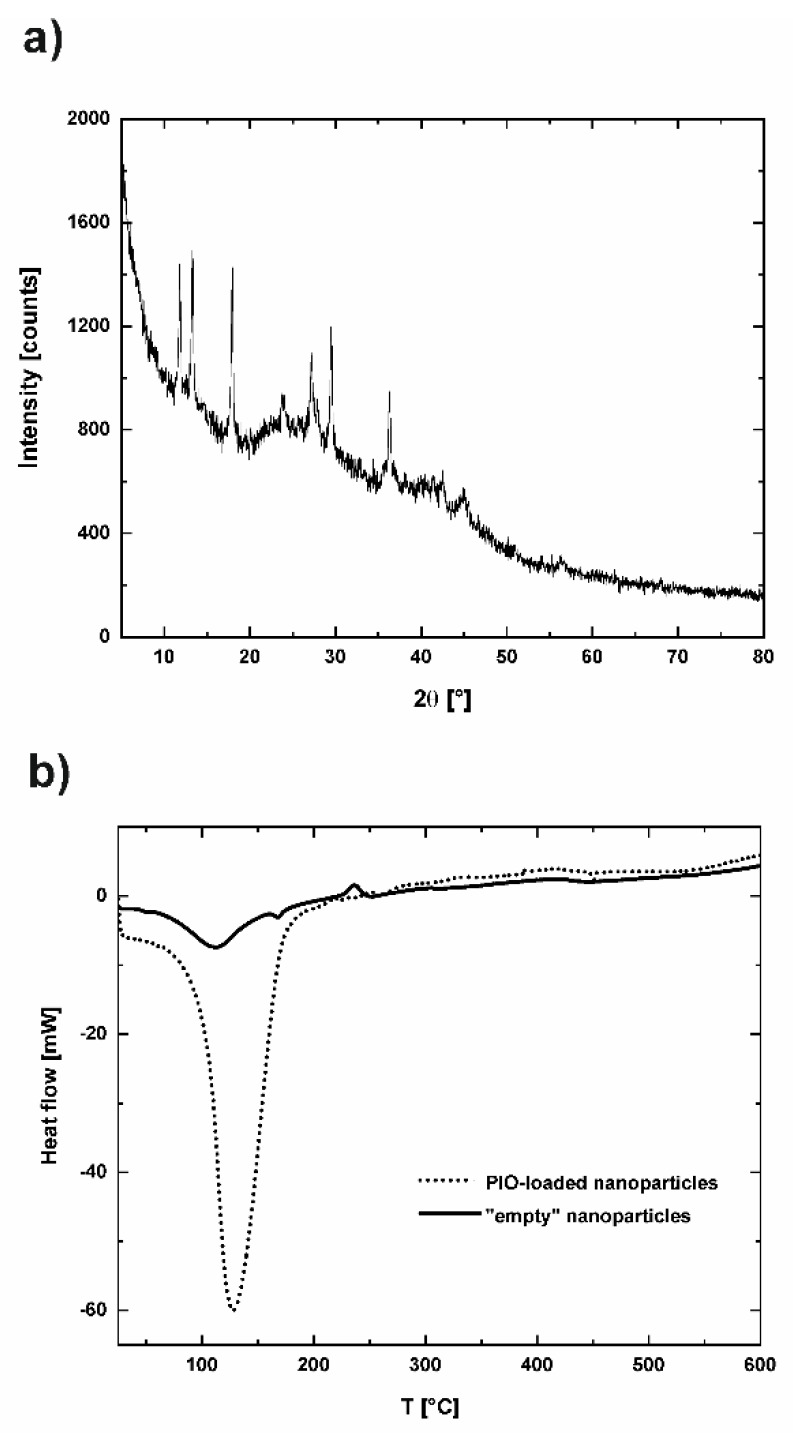
X-ray diffraction pattern for NP-PIO_B/Ch(15) nanoparticles (**a**) and differential scanning calorimetry thermograms of NP-PIO_B/Ch(15) and empty nanoparticles prepared using the same methodology, but without PIO added (**b**).

XRD analysis was employed to verify the physical state of PIO entrapped in the NP-PIO_B/Ch(15) nanoparticles. Diffraction pattern of the NP-PIO_B/Ch(15) nanoparticles is shown in Figure 6a. Amorphous character of the polymer matrix is clearly visible, with amorphous halo between 5° and 60°, with three very broad peaks at 2θ = 13.7°, 23.0°, and 40.0°, characteristic for the sodium alginate [28]. The sharp reflections at diffraction angles (2θ) of 12.2°, 13.4°, 18.0°, 23.7°, 27.6°, 29.4°, and 36.5° are characteristic for PIO [29], suggesting the crystalline character of the entrapped drug.

To understand the interations of PIO with polymer matrix the DSC analyses were performed for the NP-PIO_B/Ch(15) nanoparticles and the particles obtained with the same method, but without the drug added (empty nanoparticles). The results are presented in Figure 6b. As known from literature, the pure, crystalline PIO shows sharp endothermic peak at 197 °C, at the melting point of the drug [30]. In the DSC thermogram of NP-PIO_B/Ch(15) this peak is shifted to a much lower temperature (129 °C), which indicates its strong interaction with the polymer matrix. At the same time the intensity of the peak is high, suggesting the crystalline nature of PIO entrapped in nanoparticles, in agreement with XRD analysis. The analysis of empty particles shows three peaks characteristic for the main component of the matrix—sodium alginate: endothermic peak at 111 °C, which can be assigned to the dehydration process, another small endothermic peak at 168 °C, and stronger exothermic peak at 236 °C. The observed peaks are shifted compared to pure sodium alginate (80 °C, 200 °C, 240 °C [31]), probably as a result of the interaction between polymeric components of the matrix. First two peaks disappear in the thermogram of NP-PIO_B/Ch(15), probably due to overlapping with the drug peak of much higher intensity. The exothermic peak shifted to 217 °C and its intensity decreased considerably. These observations confirm further the strong interactions between PIO and alginate in the matrix.

### 3.4. Deposition of NP-PIO_B/Ch(15) Nanoparticles on mBNC

**Figure 7:**
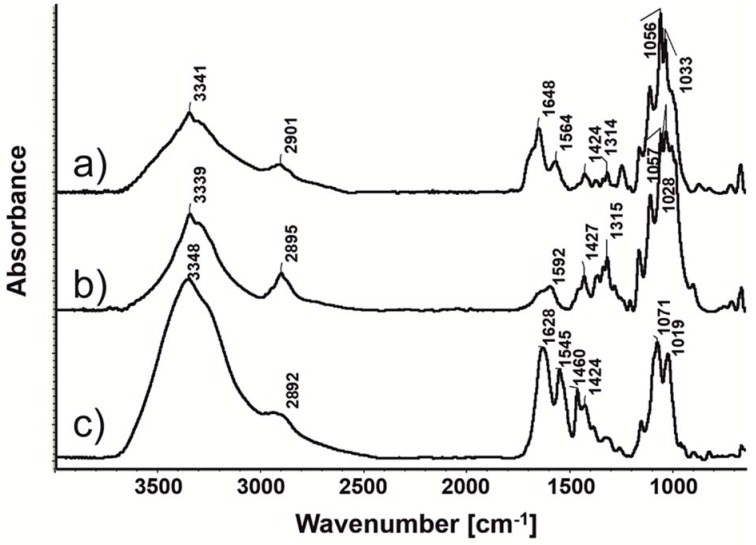
Attenuated Total Reflectance (ATR)-FTIR spectra of (a) modified bacterial nanocellulose surface with carboxyl groups (mBNC), (b) NP-PIO_B/Ch(15), and (c) mBNC-NP-PIO_B/Ch(15).

The obtained optimized drug delivery system was next tested as a bioactive component of the mBNC material with potentially application as a wound-healing dressing in the management of diabetic foot ulcers. For this purpose, the optimized nanoparticulate delivery system was covalently attached to the surface of mBNC using EDC/NHS chemistry. The concentrations of EDC and NHS necessary for activation of carboxymethyl groups of mBNC were optimized by us earlier [20]. In all the experiments EDC:NHS ratio was 1:3 and the concentrations of both compounds were adjusted to their minimal effective values (0.008 M and 0.025 M for EDC and NHS, respectively). The coupling reaction led to the formation of amide bonds between the amine groups of chitosan present in the outer shell of the particles and the carboxyl groups of mBNC, as confirmed by ATR-FTIR spectra of the unmodified mBNC sheets, PIO-containing nanoparticles (NP-PIO_B/Ch(15)) and mBNC with bound nanoparticles (mBNC-NP-PIO_B/Ch(15)) (Figure 7). The covalent attachment of the particles to the mBNC surface was confirmed by the appearance of the strong amide band I (at 1628 cm^−1^, stretching vibrations of C=O in amide group) and amide band II (1545 cm^−1^, deformation vibrations of N-H in amide group) in the mBNC-NP-PIO_B/Ch(15) sample.

**Figure 8:**
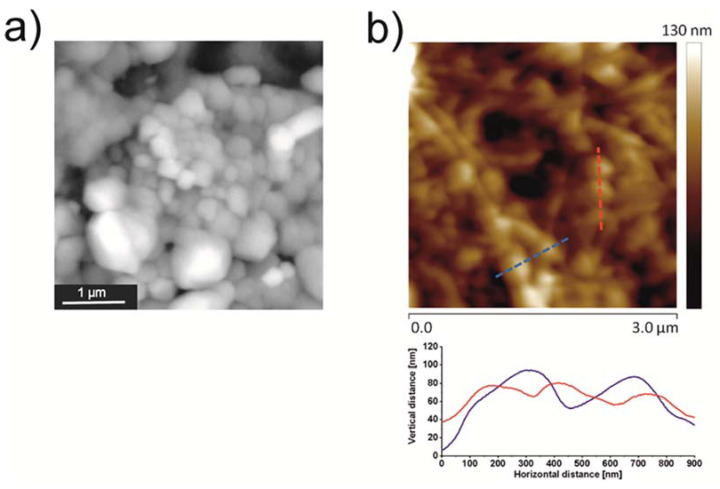
The surface of mBNC-NP-PIO_B/Ch(15): (**a**) SEM image, (**b**) AFM image (top), and a cross-section profiles (bottom) extracted from the places marked by dashed blue and red lines.

Morphology of the obtained material was visualized using SEM and AFM. SEM images revealed the presence of a large number of spherical structures of nanometric size at the surface of mBNC (Figure 8a). This observation was confirmed using AFM visualization (Figure 8b). The strands of nanoparticles decorating nanocellulose fibrils are clearly visible. The average diameter of the particles can be reasonably estimated only in the horizontal direction due to the significant pressure of the tip exerted on the soft hydrogel particle. However, the analysis of the AFM images allows to define the average size of the attached particles as being around 200 nm, which is in a good agreement with the SEM analysis, and optical microscopy results.

**Figure 9:**
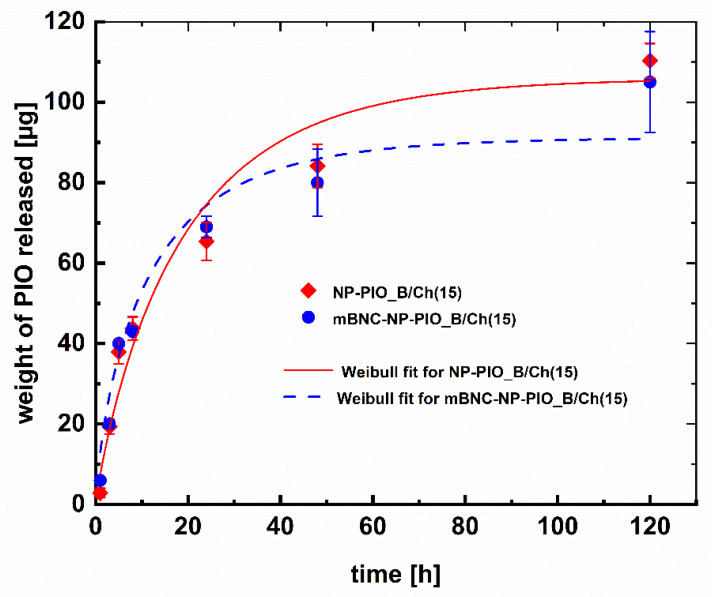
PIO release profiles from mBNC-NP-PIO_B/Ch(15) in suspension (rectangles) and bound to mBNC (NP-PIO-mBNC) (circles) to a mixture of 10 mM PBS aqueous solution (pH = 7.4) and methanol (*v*/*v* = 1:1) at 37 °C. The fitted lines present the Weibull fit to the obtained release data—the exact parameters for both curves are presented in Table 2.

The release of PIO from the mBNC-attached nanoparticles was studied and compared with the release from the unbound nanoparticles. The drug was released from the material in a controlled manner, as shown in Figure 9. The deposition process did not change the release profile of PIO. Drug loss was not observed during the deposition process. Three different kinetic models (Higuchi, Peppas, and Weibull), frequently applied to the drug release from the nanoparticulate systems, were fitted to the release profiles obtained for unbound NP-PIO_B/Ch(15) and for the mBNC-NP-PIO_B/Ch(15) material. The fitting parameters obtained for all applied models used are presented in Table 2. Both Higuchi and Peppas models are a short time approximations, so the fitting procedure was limited to the first 60% of the release profile. The Higuchi model resulted in the relatively poor approximation for both systems, therefore was not analyzed further. In the semi-empirical Peppas equation a is the kinetic constant and k exponent characterize the diffusion mechanism. Based on this model the release of PIO from the unbound particles can be described by super case II mechanism (k > 0.89), while the release from the mBNC-bound particles follows a non-Fickian mechanism (0.45 < k < 0.89) [32].

The empirical Weibull equation allows to analyze the full data set. The release mechanism may be, according to this model, described based on the d value in the Weibull equation [33]. For both NP-PIO_B/Ch(15) and mBNC_NP-PIO_B/Ch(15) systems d value was found to be in the range of 0.75–1, which is typical for a combined (Fickian diffusion and Case II transport) mechanism. Thus, Weibull model analysis leads to the conclusions which are in agreement with these obtained based on the Peppas model. The fittings of our experimental data to a Weibull model are presented in Figure 9.

### 3.5. Biological Studies

**Figure 10:**
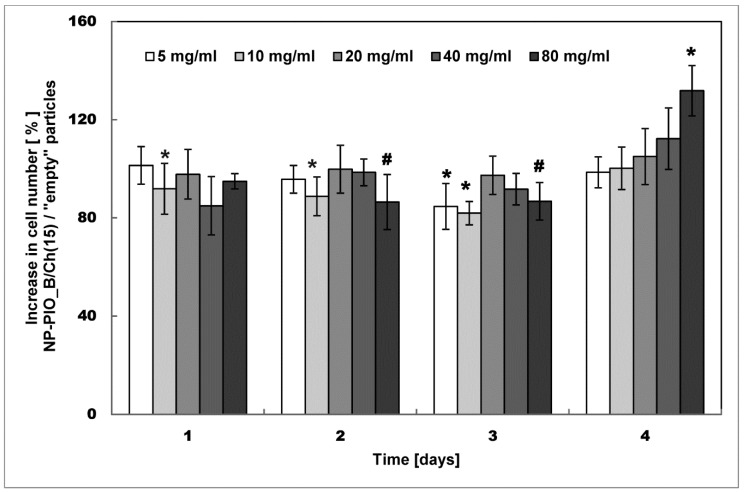
Cell proliferation results for 3T3-L1 (Mouse Embryonic Fibroblasts (MEF)) cells grown in the presence of the NP-PIO_B/Ch(15) and empty nanoparticles (prepared with the same methodology but without PIO added) studied on day 1, 2, 3, and 4 of the culturing. Statistical significance was calculated using the Mann–Whitney test comparing the groups of NP-PIO_B/Ch(15) and empty particles for the five tested concentrations of nanoparticles. * indicates statistical significance between adequate materials (*p* < 0.05); **#** indicates statistical significance between adequate materials (*p* < 0.07).

The influence of the optimized PIO delivery system (NP-PIO_B/Ch(15) nanoparticles) on the proliferation of fibroblasts was also tested. The results are shown in Figure 10. For majority of used concentrations, the relative proliferation changes over time following a similar pattern, not in the obvious way. At the beginning (days 1–3), proliferation is inhibited, and on the day 4 we observed the opposite effect, cell growth. The effect increases with concentration and at 80 mg/mL we have observed an increase of up to 130%. It can be assumed that this is a combination of two opposite proliferation moderating effects that differ in kinetics. This may be also related to the release kinetics of pioglitazone or a feature of its biological activity. Literature reports show inhibition [34] or acceleration [11] of cell proliferation by this compound depending on the circumstances and incubation time. Such time depending variability of proliferation-related properties may involve interaction of compound with growth factors as it is in the case of polycations [35].

**Figure 11:**
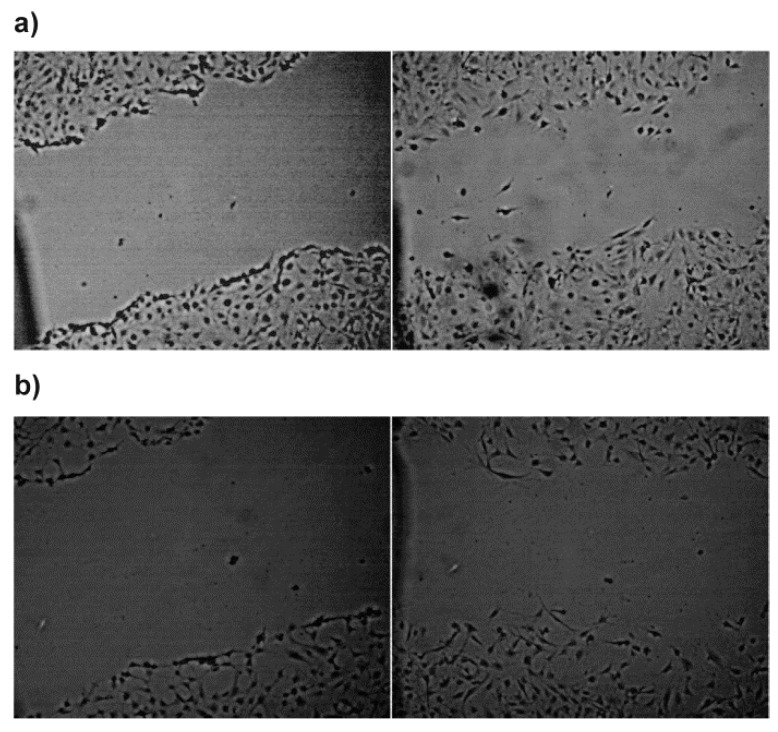
Simulated wound healing ex vivo test-scratch assay for 3T3-L1 (MEF) cells in the presence of (**a**) NP-PIO_B/Ch(15) nanoparticles and (**b**) empty nanoparticles (prepared with the same methodology but without PIO added). Left panels illustrate the fresh scratch in a cell monolayer after 2 days of culturing while right panels show the same scratch in a cell monolayer after 11 h of further incubation with the respective nanoparticles.

To study the influence of NP-PIO_B/Ch(15) system on the fibroblasts motility and wound-healing properties the ex vivo “scratch“ assay was performed (see Figure 11). The scratch was made in the confluent monolayer of 3T3-L1 fibroblasts, and the effect of PIO-loaded and empty nanoparticles on the fibroblasts migration to close the scratch. In the case of PIO-loaded nanoparticles, the cells, in addition to propagating on the edges of the scratch, were also effectively migrating into the simulated wound interior. This effect was observed in the case of more than 75% repetitions for PIO-loaded, and only 25% for empty nanoparticles. This observation suggests that the system may have a positive effect on wound healing process. 

## 4. Conclusions

We have successfully entrapped PIO in the ALG/HPC hydrogel nanoparticles. The composition of the system was optimized. The addition of chitosan to the crosslinking solution increased the control over the PIO release profile, allowing for the slow, prolonged release up to 5 days. The presence of chitosan in the outer shell of particles enabled a covalent bonding of the particles to the surface of mBNC, without any significant loss of entrapped PIO, or change in its release profile. Proliferation tests showed that, after initial inhibition, on day 4 the NP-PIO_b/Ch(15) nanoparticles stimulated proliferation of fibroblasts in a dose-dependent manner, while the scratch assay revealed the higher motility of fibroblasts into the simulated wound interior in the presence of this nanoparticulate system. The obtained bioactive hybrid, microstructured material can be potentially applied as a wound dressing, useful in the skin ulcer treatment. We believe it may be of special interest in the management of foot ulcers in diabetic patients.

## Figures and Tables

**Table 1:** Composition of the reaction mixture and properties of the obtained particulate systems.

Sample	ALG:HPC (*w*/*w*)	Surfactant Used	Chitosan in Crosslin King Medium	C _PIO_ [mg/mL]	d_av_ * [µm]	LE [%]	EE [%]	Time of 50% Release [h]
NP-PIO_A	3:1	Pluronic^®^ P103	NO	0.10	1.10	0.50 ± 0.03	43.20 ± 1.63	25
NP-PIO_A/Ch	3:1	Pluronic^®^ P103	YES	0.10	0.95	1.04 ± 0.02	72.85 ± 1.51	47
NP-PIO_B	4:1	Pluronic^®^ P103	NO	0.10	1.40	0.80 ± 0.03	56.82 ± 2.65	24
NP-PIO_B/Ch	4:1	Pluronic^®^ P103	YES	0.10	0.20	1.10 ± 0.01	77.36 ± 1.02	45
NP-PIO_B/Ch(15)	4:1	Pluronic^®^ P103	YES	0.15	0.15	1.35 ± 0.04	83.91 ± 2.68	25
NP-PIO_B/Ch(15T)	4:1	Tween^®^ 85	YES	0.15	0.70	1.32 ± 0.06	86.34 ± 2.06	24

* obtained from histograms created based on SEM photographs (weighted average).

**Table 2:** Parameters obtained by fitting different release kinetic models to experimental release profiles of PIO for unbound and mBNC-bound nanoparticles.

System	NP-PIO_B(15)	mBNC-NP-PIO_B(15)
Higuchi: $y=a\sqrt{x}$		
a	11.56	15.15
R^2^	0.679	0.865
Peppas: $y={\mathrm{ax}}^{k}$		
a	5.56	10.14
k	1.04	0.719
R^2^	0.939	0.917
Weibull: $y=a-\left(a-b\right)e^{{\left(-\mathrm{kx}\right)}^{d}}$		
a	105.92	91.23
b	0.00	0.00
k	0.05	0.08
d	0.88	0.76
R^2^	0.961	0.898
